# Correlation between cardiometabolic index and psoriasis: a cross-sectional analysis using NHANES data

**DOI:** 10.3389/fphys.2025.1552269

**Published:** 2025-03-18

**Authors:** Mengxue Li, Yixiao Gan, Hong Cheng, Zhicheng Wang

**Affiliations:** Department of Transfusion Medicine, Huashan Hospital, Fudan University, Shanghai, China

**Keywords:** cardiometabolic index, psoriasis, cross-sectional study, NHANES, multivariate logic analysis

## Abstract

**Background:**

Psoriasis is closely associated with metabolic health. The Cardiometabolic Index (CMI) is an innovative and easily obtainable metric employed to assess cardiometabolic health. This study aims to examine the possible relationship between CMI and psoriasis.

**Methods:**

Data from four successive cycles of the National Health and Nutrition Examination Survey (NHANES) conducted between 2003–2004 and 2009–2014 were employed. This encompassed adults with self-reported psoriasis diagnoses and comprehensive information necessary for calculating the CMI. The calculation formula for CMI is Triglycerides (TG)/High-density lipoprotein cholesterol (HDL-C) × WHtR (WHtR = waist circumference/height). A multivariable logistic regression model was utilized to examine the linear relationship between CMI and psoriasis. Subgroup analyses were conducted to investigate potential contributing factors. The linear relationship was further established using smooth curve fitting.

**Results:**

This study, utilizing NHANES data, comprised a cohort of 7,327 American adults. The multivariable logistic regression analysis indicated that in the fully adjusted model, people with the greatest CMI had a 71% increased probability of psoriasis relative to those with the lowest CMI (OR = 1.71; 95% CI, 1.11–2.61, P < 0.05). Smooth curve fitting demonstrated a linear connection between CMI and psoriasis (P < 0.05). The subgroup analysis revealed no significant interactions between CMI and specific subgroups (all interactions P > 0.05).

**Conclusion:**

Our research indicates a substantial linear correlation between CMI and psoriasis in American adults. This method facilitates the identification of groups at increased risk for psoriasis, therefore guiding therapeutic solutions and public health activities to improve metabolic and dermatological health outcomes.

## Background

Psoriasis, a common chronic skin disorder, impacts around 60 million people worldwide, encompassing both adults and children. This condition is characterized by the emergence of erythematous plaques adorned with silvery-white scales (Griffiths et al., 2021). With a prevalence of approximately 2%–3% among the worldwide population, psoriasis imposes a considerable socioeconomic burden (Ghoreschi et al., 2021; Armstrong et al., 2021). The pathophysiology of psoriasis is not well understood, requiring complex interactions among genetic factors, immune system abnormalities, and environmental stimuli (Armstrong and Read, 2020).

Recent years have witnessed considerable research interest in the correlation between metabolic problems and psoriasis. A prospective cohort study has demonstrated a positive association between triglyceride (TG) levels and the risk of developing psoriasis (Xiao et al., 2022). Mendelian randomization studies provide more evidence for this relationship, indicating that increased triglyceride levels may serve as a causative risk factor for the disease (Zhang et al., 2023; Greve et al., 2024; Zhou Q. et al., 2024). A cross-sectional study has demonstrated a strong positive connection between the triglyceride glucose (TyG) index and psoriasis (Huang et al., 2023). The aggregated results of this research underscore a notable correlation between dyslipidemia and psoriasis, establishing lipid profiles as both easily accessible indicators and potentially potent biomarkers for the condition.

In clinical environments, the diagnosis of lipid metabolism problems generally necessitates a combination of anthropometric evaluations and certain biochemical indicators. Researchers have utilized the Cardiometabolic Index (CMI), an essential metric that integrates obesity indicators such as the waist-to-height ratio (WHtR) and the triglyceride to high-density lipoprotein cholesterol (TG/HDL-C) ratio, to identify diabetes and obesity (Wakabayashi and Daimon, 2015). Comprehensive research has validated the CMI’s effectiveness in forecasting and diagnosing a range of illnesses, including as non-alcoholic fatty liver disease, hypertension, chronic kidney disease, depression, and endometriosis (Wang et al., 2018; Wang et al., 2024; Zhou X. et al., 2024; Guo et al., 2024; Zou et al., 2022). CMI, necessitating only basic anthropometric measurements and lipid profiles, offers a more accessible method for illness evaluation.

There is currently no research exploring the relationship between the CMI and psoriasis. In this context, our study utilized data from the National Health and NHANES for the years 2003–2004 and 2009–2014 to do a cross-sectional analysis. The main objective was to examine the potential association between CMI and psoriasis.

## Methods

### Study population

The National Center for Health Statistics (NCHS) executed the comprehensive NHANES program to assess the health and nutritional status of persons in the United States. Informed consent was obtained from all participants before to conducting interviews and tests, and the NCHS Ethical Review Committee approved the data collection techniques.

**FIGURE 1:**
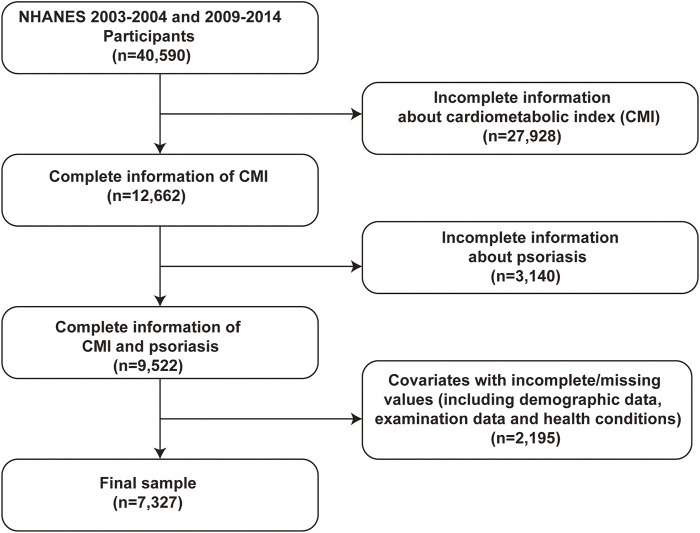
Diagram of participant enrollment process.

Participant recruitment for this study occurred throughout two intervals: 2003–2004 and 2009–2014, culminating in the enrollment of 40,590 participants. The exclusion criteria were as follows: 1) the absence of comprehensive data for computing the Cardiometabolic Index (CMI), 2) insufficient data pertaining to psoriasis, and 3) the absence of demographic, examination, or health condition data. Figure 1 displays a flowchart that delineates the participant selection procedure.

### Assessment of psoriasis

Psoriasis was identified based on participants’ affirmative responses to the queries, “Have you ever been told by a healthcare provider that you had psoriasis?” or “Have you ever been told by a doctor or other healthcare professional that you had psoriasis?”. The study excluded individuals who either declined to answer or expressed uncertainty about their diagnosis (Ma et al., 2024).

### Definition of cardiometabolic index (CMI)

CMI was utilized as a continuous variable in this research. The subsequent formula is employed to compute CMI: CMI = WHtR × [TG (mg/dL)/HDL-C (mg/dL)], WHtR = waist circumference (cm)/height (cm).

### Covariates

The analysis accounted for various clinically significant factors to determine the independent connection between the Cardiometabolic Index (CMI) and psoriasis. These encompassed sociodemographic variables and health problems. This investigation examined the factors of age, race, marital status, educational attainment, poverty income ratio (PIR), smoking habits, and alcohol intake. Smoking status was determined by lifetime cigarette consumption, categorizing non-smokers as individuals who have smoked fewer than 100 cigarettes and smokers as those who have smoked more than 100 cigarettes. Alcohol consumption was classified according to the quantity of alcoholic beverages consumed during the last 12 months, with non-consumers defined as individuals who had less than 12 and consumers as those who had 12 or more.

Trained personnel at the Mobile Examination Center (MEC) conducted anthropometric measurements, including body mass index (BMI), height, and waist circumference. The Body Mass Index (BMI) is determined by dividing an individual’s weight in kilograms by the square of their height in meters. Key health indicators such as heart attack, stroke, coronary artery disease, angina, high-density lipoprotein cholesterol (HDL-C) levels, and triglyceride levels were evaluated. Cholesterol level serum samples were handled and analyzed at the University of Minnesota in accordance with the NHANES Laboratory Procedures Manual. Participants provided self-reported data through questionnaires, detailing instances of heart attack, coronary heart disease, stroke, and angina.

### Statistical analysis

The National Health and Nutrition Examination Survey (NHANES) utilizes a complex, multi-stage sampling process in accordance with the rules established by the Centers for Disease Control and Prevention (CDC). We performed comparative analyses between psoriasis-positive and psoriasis-negative groups, employing t-tests for continuous variables and chi-square tests for categorical data. We expressed continuous variables as the mean and standard deviation, and represented categorical data as frequencies and percentages.

We utilized multivariable regression models to examine the correlation between CMI and psoriasis. We developed three logistic regression models to address different confounding variables. Model 1 was unadjusted, Model 2 was adjusted for age, gender, and race, while Model 3 was fully adjusted. Then we classified CMI values into quartiles for sensitivity analysis to evaluate the robustness of our findings. After that, we transformed CMI into logarithmic values and subsequently utilized smoothed curve analysis to clarify the relationship between CMI and psoriasis. In the end, we performed subgroup studies employing stratified multifactorial regression analysis to evaluate the stability of the connection between CMI and psoriasis. Statistical analyses were conducted using R (version 4.2.1) and EmpowerStats (version 2.0), with a significance threshold established at P < 0.05 to ascertain statistical significance.

## Results

### Baseline characteristics of the population

This research study enrolled a total of 7,327 participants based on the established inclusion and exclusion criteria. The study diagnosed 204 patients with psoriasis, yielding a prevalence rate of 2.78%. The average age of the participants was 47.44 ± 17.29 years, with 14.82% classified as Mexican American, 8.50% as non-Hispanic White, 47.44% as non-Hispanic Black, 19.34% as other Hispanic, and 9.89% as other ethnicities. The gender distribution was quite uniform, with 49.30% males and 50.70% females. The cohort’s average BMI was 28.95 ± 6.78 kg/m^2^, and the average waist circumference was 98.95 ± 16.34 cm.

Table 1 enumerates the clinical characteristics of the participants, utilizing psoriasis as a stratifying variable to categorize the population into non-psoriasis and psoriasis groups. This study reveals significant differences between people with psoriasis and those without. In comparison to those without psoriasis, individuals with psoriasis were older, more frequently identified as non-Hispanic White, more likely to be married, and exhibited a higher poverty income ratio (PIR). Psoriasis sufferers had elevated BMI, CMI, and waist circumference values in comparison to non-psoriasis subjects. Moreover, patients with psoriasis demonstrated higher prevalence rates of angina, coronary heart disease, and heart attack compared to non-psoriasis subjects, as shown in Table 1.

**TABLE 1:** Baseline characteristics of patients with and without psoriasis.

Characteristics	Overall	Psoriasis		P-value
		Without	With	
	n = 7327	n = 7123	n = 204	
Age, years	47.44 ± 17.29	47.34 ± 17.30	50.83 ± 16.39	0.003
Gender, n (%)				0.936
Male	3612 (49.30%)	3512 (49.31%)	100 (49.02%)	
Female	3715 (50.70%)	3611 (50.69%)	104 (50.98%)	
Race, n (%)				<0.001
Mexican American	1086 (14.82%)	1065 (14.95%)	21 (10.29%)	
Other Hispanic	623 (8.50%)	608 (8.54%)	15 (7.35%)	
Non-Hispanic White	3476 (47.44%)	3348 (47.00%)	128 (62.75%)	
Non-Hispanic Black	1417 (19.34%)	2144 (30.10%)	65 (31.86%)	
Other Race	725 (9.89%)	1748 (24.54%)	49 (24.02%)	
Education level, n (%)				0.983
Less than 9th	630 (8.60%)	614 (8.62%)	16 (7.84%)	
9-11th	1074 (14.66%)	1044 (14.66%)	30 (14.71%)	
High school	1617 (22.07%)	1573 (22.08%)	44 (21.57%)	
Some college	2209 (30.15%)	2144 (30.10%)	65 (31.86%)	
College graduate	1797 (24.53%)	1748 (24.54%)	49 (24.02%)	
Marital status, n (%)				0.015
Married	3821 (52.15%)	3716 (52.17%)	105 (51.47%)	
Never married	1411 (19.26%)	1385 (19.44%)	26 (12.75%)	
Others	2095 (28.59%)	2022 (28.39%)	73 (35.78%)	
Family PIR	2.52 ± 1.65	2.52 ± 1.64	2.65 ± 1.77	0.493
BMI, kg/cm^2^	28.95 ± 6.78	28.91 ± 6.76	30.47 ± 7.30	0.001
HDL (mg/dL)	53.63 ± 15.77	53.66 ± 15.71	52.49 ± 17.47	0.096
TG (mg/dL)	130.59 ± 117.59	130.26 ± 117.57	142.07 ± 117.94	0.062
Height, cm	168.03 ± 10.01	168.05 ± 10.02	167.42 ± 9.60	0.468
Wasit circumference, cm	98.95 ± 16.34	98.83 ± 16.31	103.27 ± 16.86	<0.001
CMI	1.80 ± 2.51	1.78 ± 2.48	2.25 ± 3.44	0.013
Coronary heart disease, n (%)				0.008
Yes	257 (3.51%)	243 (3.41%)	14 (6.86%)	
No	7070 (96.49%)	6880 (96.59%)	190 (93.14%)	
Angina, n (%)				0.009
Yes	164 (2.24%)	154 (2.16%)	10 (4.90%)	
No	7163 (97.76%)	6969 (97.84%)	194 (95.10%)	
Heart attack, n (%)				<0.001
Yes	248 (3.38%)	231 (3.24%)	17 (8.33%)	
No	7079 (96.62%)	6892 (96.76%)	187 (91.67%)	
Stroke, n (%)				0.516
Yes	230 (3.14%)	222 (3.12%)	8 (3.92%)	
No	7097 (96.86%)	6901 (96.88%)	196 (96.08%)	
Smoking, n (%)				0.066
Yes	3273 (44.67%)	3169 (44.49%)	104 (50.98%)	
No	4054 (55.33%)	3954 (55.51%)	100 (49.02%)	
Drinking, n (%)				0.383
Yes	5409 (73.82%)	5253 (73.75%)	156 (76.47%)	
No	1918 (26.18%)	1870 (26.25%)	48 (23.53%)	

The mean ± standard deviation for continuous variables was calculated, and the p-value was obtained by a weighted linear regression model. % for categorical variables, the p-value for categorical variables was calculated using a weighted chi-square test. The Poverty Income Ratio (PIR) is the ratio of family income to the poverty threshold. Body Mass Index (BMI). High-density lipoprotein cholesterol (HDL-C). TG, triglycerides. Cardiometabolic Index (CMI).

### Association between CMI and psoriasis

Table 2 displays the results of the multivariate logistic regression analysis using the three models. The logistic regression investigation revealed a strong connection between CMI and psoriasis in both model 1 and model 2. The positive correlation remained consistent in the completely corrected model 3 (OR = 1.04, 95% CI: 1.00–1.07, P = 0.0447). We noted that for each unit rise in CMI, there was a 4% increased likelihood of psoriasis prevalence. Our subsequent analyses, converting the continuous variable CMI into a categorical variable, continued to demonstrate statistically significant outcomes. Moreover, individuals in the highest quartile of CMI had a 71% greater prevalence of psoriasis compared to those in the lowest quartile of CMI (OR = 1.71, 95% CI: 1.11–2.61).

**TABLE 2:** Association between CMI and psoriasis.

CMI class	Model 1 OR (95%CI) P value	Model 2 OR (95%CI) P value	Model 3 OR (95%CI) P value
Continuous	1.04 (1.01, 1.07) 0.0135	1.04 (1.00, 1.07) 0.0276	1.04 (1.00, 1.07) 0.0447
Q1	1.0	1.0	1.0
Q2	1.31 (0.85, 2.02) 0.2276	1.26 (0.82, 1.95) 0.2944	1.25 (0.81, 1.94) 0.3118
Q3	1.42 (0.93, 2.17) 0.1091	1.39 (0.90, 2.14) 0.1363	1.36 (0.88, 2.11) 0.1641
Q4	1.84 (1.23, 2.77) 0.0033	1.74 (1.14, 2.63) 0.0097	1.71 (1.11, 2.61) 0.0141

Model 1: no covariates were adjusted. Model 2: age, gender, and race were adjusted. Model 3: age, gender, race, marriage, education level, PIR, smoking, alcohol consumption, coronary heart disease, angina, heart attack, and stroke were adjusted. PIR, ratio of family income to poverty; BMI, body mass index; CMI, cardiometabolic index.

**FIGURE 2:**
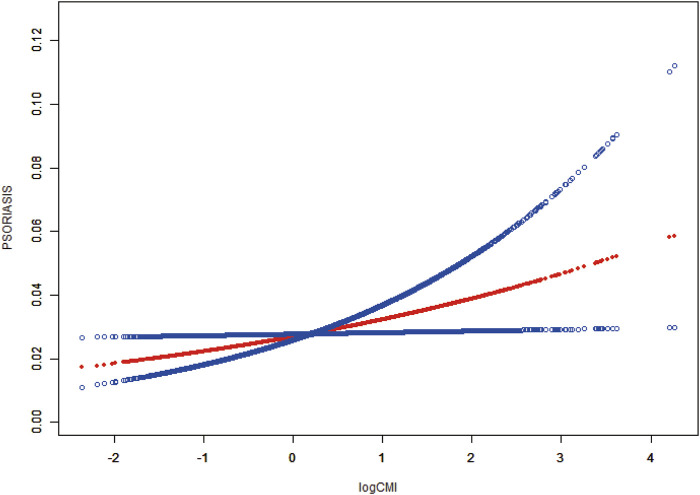
The association between CMI and psoriasis. The smoothed curve fit between the variables is shown as a solid red line, with the 95% CIs of the fitted results indicated by the blue line. Controlled attenuation parameter. CMI, cardiometabolic index.

We conducted smooth curve fitting (Figure 2) to elucidate the relationship between CMI and psoriasis. The findings demonstrated a linear correlation between CMI and psoriasis (P for overall < 0.05, P for nonlinear and log-likelihood ratio tests >0.05).

### Subgroup analyses

**FIGURE 3:**
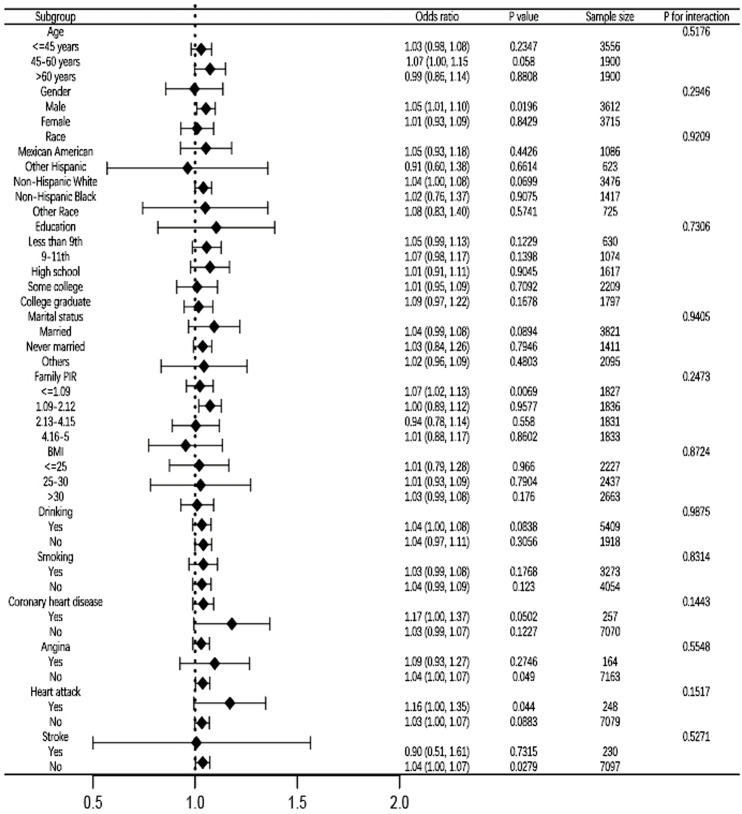
Subgroup analysis for the association between CMI and psoriasis fully adjusted. In the subgroup analyses, the models were not tuned for the stratification variables themselves. PIR, ratio of family income to poverty. BMI, body mass index. CMI, cardiometabolic index.

To evaluate the stability of the association between CMI and psoriasis while accounting for the influence of additional variables, we performed subgroup analyses based on age, gender, race, education level, marital status, family PIR, BMI, alcohol consumption, smoking, coronary heart disease, angina, myocardial infarction, and stroke. None of these factors influenced the favorable correlation between CMI and infertility, as illustrated in Figure 3. With each unit rise in CMI, the likelihood of psoriasis prevalence increased by 5% in male patients (OR = 1.05, 95% CI: 1.01–1.10). Individuals with a family PIR below 1.09 have a 7% increased likelihood of psoriasis prevalence for each unit increase in CMI (OR = 1.07, 95% CI: 1.03–1.13). In the absence of angina, the probabilities of psoriasis prevalence increase by 4% for each unit rise in CMI (OR = 1.04, 95% CI: 1.00–1.07). With each unit rise in CMI, the likelihood of psoriasis prevalence increased by 16% in patients with a history of heart attack (OR = 1.16, 95% CI: 1.00–1.35). In the absence of stroke, the odds of psoriasis prevalence rise by 4% for each unit increase in CMI (OR = 1.04, 95% CI: 1.00–1.07).

## Discussion

This cross-sectional study with 7,327 subjects revealed a new link between CMI levels and psoriasis prevalence in American adults. Whether CMI is evaluated as a continuous or categorical variable, the findings demonstrated a linear connection between CMI and psoriasis. The study further validated the positive correlation between elevated CMI levels and the occurrence of psoriasis through smoothed curve fitting. This discovery corroborates and expands the initial hypothesis of the study, emphasizing the crucial role of CMI in psoriasis and offering important insights into the role of lipid metabolism in the progression of this disorder. This study is the inaugural investigation on the correlation between CMI and the likelihood of developing psoriasis.

Lipid metabolism’s role in psoriasis has attracted considerable attention from researchers, as indicated by recent studies (Nowowiejska et al., 2021; Nowowiejska et al., 2023; Seiringer et al., 2024). The CMI is a crucial parameter for defining metabolic profiles, considering both abdominal obesity and serum lipid levels (Miao et al., 2023). Our data indicate a significant association between increased CMI values and the incidence of psoriasis. The infiltration of activated macrophages and T lymphocytes into abdominal visceral adipose tissue induces adipocytes to release nonesterified fatty acids (NEFA) and to synthesize various adipokines and proinflammatory chemicals. This discharge may induce chronic, low-grade inflammation and non-alcoholic fatty liver disease (NAFLD) (Wolk and Sabat, 2016). NAFLD makes insulin resistance worse in the body and the liver. It also causes atherogenic dyslipidemia and sets off a chain of pro-inflammatory, pro-coagulant, pro-oxidant, and profibrogenic mediators. These mediators are crucial in the pathophysiology of psoriasis. Furthermore, hepatic mediators originating from steatotic and inflammatory livers may intensify psoriasis severity by promoting keratinocyte proliferation, inflammation, and the upregulation of vascular adhesion molecules (Mantovani et al., 2016).Due to the strong association between CMI and psoriasis, it is essential in clinical practice to thoroughly assess individuals exhibiting elevated CMI levels. Implementing effective strategies to mitigate these risk factors is essential for reducing the likelihood of acquiring psoriasis.

Recent academic research has emphasized the strong association between obesity and dyslipidemia. Obesity is often characterized by a body mass index (BMI) over 30 kg/m^2^, according to various clinical criteria (Caballero, 2019). A cross-sectional investigation has demonstrated a roughly linear association between BMI and the risk of dyslipidemia in Chinese individuals (Zheng et al., 2024). This link is additionally corroborated by another cross-sectional study, which illustrates that the prevalence of dyslipidemia in the pediatric and teenage population is favorably associated with elevated BMI values (Reuter et al., 2016). A thorough population-based investigation has revealed that elevated BMI in nursing mothers is substantially correlated with an increased risk of hypercholesterolemia, hypertriglyceridemia, hypo-HDL-cholesterolemia, hyper-LDL-cholesterolemia, and dyslipidemia (Yu et al., 2022). These studies jointly highlight the significance of obesity in the evaluation and treatment of dyslipidemia among many demographic groups.

The study establishes a significant correlation between increased CMI levels and the occurrence of psoriasis, a relationship that remains statistically significant despite controlling for several potential confounding variables. This persistent relationship indicates a complicated interaction between obesity and dyslipidemia concerning psoriasis. Extensive prior research has elucidated the associations between these metabolic disorders and psoriasis. Extensive systematic reviews and meta-analyses have repeatedly demonstrated strong associations between psoriasis and dyslipidemia as well as obesity. Individuals with psoriasis exhibit elevated rates of obesity and a greater propensity for dyslipidemia relative to the general population (Alshahrani et al., 2023; Mirghani et al., 2023). Research suggests that obesity and dyslipidemia can synergistically exacerbate skin inflammation linked to psoriasis, clarifying how metabolic problems may contribute to the initiation and worsening of psoriatic inflammation. This highlights the urgent necessity for rigorous care of coexisting metabolic diseases in individuals with psoriasis (Ikeda et al., 2022).

Previous studies in psoriasis research have predominantly investigated the relationships among dietary consumption, environmental exposures, inflammation, and the onset of psoriasis (Song et al., 2024; Chen et al., 2023). Conversely, our research evaluates a wider range of lipid metabolism variables, incorporating them into the CMI. This method offers a more thorough and direct way to evaluate the metabolic factors linked to psoriasis. Moreover, our analysis indicates that the outcomes are uniform across various subgroups. Current cross-sectional investigations rarely emphasize the consistency of the intervention effects across all subgroups, which enhances our trust in the findings. This uniformity is essential since it improves the trustworthiness of our conclusions and their applicability across diverse patient populations. Consequently, it is imperative to emphasize and proactively address metabolic health in the prevention and early intervention of psoriasis.

### Study strengths and limitations

This study offers an innovative investigation into the correlation between the CMI and psoriasis, employing cross-sectional data from the NHANES database. Prior studies have not thoroughly investigated the correlation between CMI and psoriasis, and this study seeks to identify CMI as a potential predictor of psoriasis. We employed multivariate logistic regression analysis and subgroup analysis to clarify the relationship between CMI and psoriasis and to evaluate the reproducibility of our results. These findings are significantly relevant for formulating strategies aimed at the prevention and early intervention of psoriasis.It is essential to recognize the limitations inherent in our research. The cross-sectional design of this study restricts our capacity to determine a causal relationship between CMI and psoriasis, as it permits the analysis of correlation alone at one specific moment. The study aimed to provide reference values for the early detection of psoriasis, rather than to prove causality. Secondly, our findings pertain only to the American population and may not be applicable to other countries or areas, underscoring the necessity for additional study to assess the global relevance of our results. The dependence on self-reported questionnaire data to ascertain the presence or absence of psoriasis may result in misdiagnoses or an underestimate of cases, especially for mild or atypical manifestations of the condition. Recall bias may also affect the validity of our study’s results. In the end, the NHANES database can’t tell the difference between different types of psoriasis or give information about the different stages of disease development. This could affect how different types and stages of psoriasis interact biochemically with CMI.

## Conclusion

Our research demonstrates a linear correlation between the CMI and psoriasis within the American adult demographic. However, further extensive, prospective investigations are necessary to corroborate the findings of this research. This research would be crucial in identifying groups at increased risk for psoriasis, thereby informing clinical practices and public health measures to improve metabolic and dermatological health outcomes.

## Data Availability

The original contributions presented in the study are included in the article/supplementary material, further inquiries can be directed to the corresponding author.
